# Triple-negative apocrine carcinoma as a rare cause of a breast lump in a Syrian female: a case report and review of the literature

**DOI:** 10.1186/s12905-021-01539-3

**Published:** 2021-11-25

**Authors:** Sawsan Ismail, Haidara Kherbek, Jana Skef, Nadim Zahlouk, Rafik Abdulal, Zuheir Alshehabi

**Affiliations:** 1 Department of Pathology, Faculty of Medicine, Tishreen University, Lattakia, Syria; 2 Cancer Research Center, Tishreen University, Lattakia, Syria; 3 Faculty of Medicine, Tishreen University, Lattakia, Syria; 4 Department of Oncology, Tishreen University Hospital, Lattakia, Syria; 5 Department of General Surgery, Al Nada Surgical Hospital, Lattakia, Syria; 6 Department of Pathology, Cancer Research Center, Tishreen University, Lattakia, Syria

**Keywords:** Apocrine carcinoma, Triple-negative apocrine carcinoma, Apocrine differentiation, Immunohistochemistry, Breast Cancer

## Abstract

**Background:**

Apocrine carcinoma is a rare tumor that constitutes < 4% of all breast malignancies, characterized by the proliferation of large atypical cells with strictly defined borders, abundant eosinophilic cytoplasm, large nuclei, and prominent nucleoli in more than 90% of tumor cells. Triple-negative apocrine carcinoma is a rare molecular subtype that constitutes less than 1% of triple-negative breast cancers and is characterized by negative expression of estrogen receptor, progesterone receptor, and human epidermal growth factor receptor, with positive expression of androgen receptor.

**Case presentation:**

We report a case of a 45-year-old Syrian female who presented to our hospital due to a painless palpable mass in her left breast. Following physical and radiological examinations, an excisional biopsy was performed. Microscopic examination of the specimen followed by immunohistochemical staining revealed the diagnosis of a triple-negative apocrine carcinoma.

**Conclusion:**

Triple-negative apocrine carcinoma is an extremely rare neoplasm that must be considered in the differential diagnoses of breast lesions through detailed clinical, histological, and immunohistochemical correlations. In our manuscript, we aimed to present the first case report of a Syrian female who was diagnosed with a triple-negative apocrine carcinoma, aiming to highlight the importance of detailed clinical, histological and immunohistochemical correlations with a detailed review of diagnostic criteria, molecular characteristics, and treatment recommendations.

## Background

Apocrine carcinoma (AC) is a rare tumor that constitutes less than 4% of all breast malignancies, characterized by the proliferation of large atypical cells with strictly defined borders, abundant eosinophilic cytoplasm, large nuclei, and prominent nucleoli in more than 90% of tumor cells [1]. Apocrine carcinomas carry a distinct immunohistochemical profile represented by the negative expression of estrogen receptor (ER) and progesterone receptor (PR), with positive expression of androgen receptor (AR) and gross cystic disease fluid protein-15 (GCDFP-15). Human epidermal growth factor receptor-2 (HER2/neu) overexpression is present in approximately 50% of apocrine carcinoma cases [2], whereas HER2-negative apocrine carcinomas are classified as triple-negative apocrine carcinomas (TNACs) which are extremely rare neoplasms constituting less than 1% of all triple-negative breast cancers (TNBCs) [3]. Although TNBCs have a poor prognosis due to the negative expression of hormone receptors and the inability to apply targeted therapy, triple-negative apocrine carcinomas were found to have a better prognosis and overall survival, which highlights the importance of diagnosing and distinguishing this rare subtype [4]. Herein, we present the first case report from Syria of a 45-year-old female who was diagnosed with a triple-negative apocrine carcinoma.

## Case presentation

**Fig. 1:**
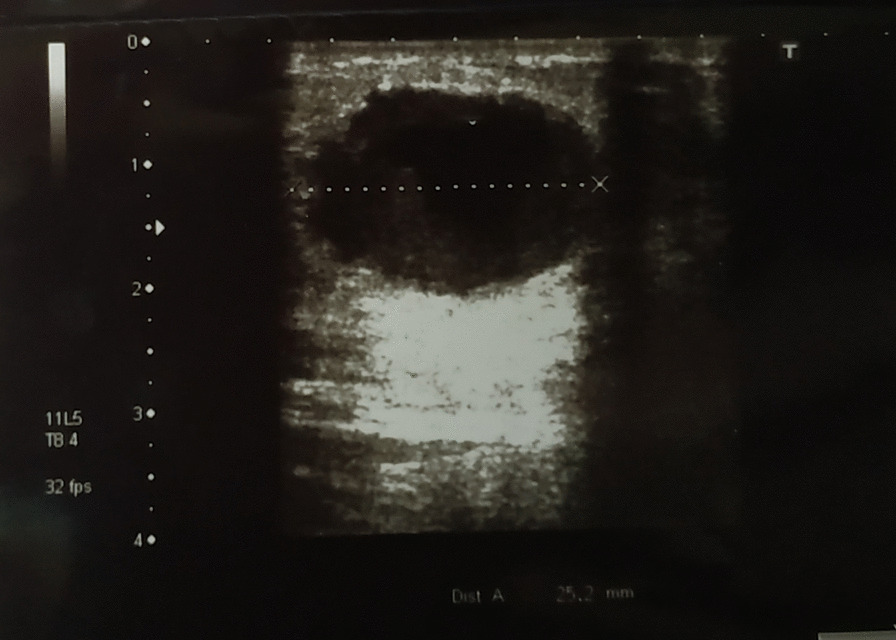
Ultrasonography of the breast demonstrating a well-defined hypoechoic cyst with heterogeneous components

**Fig. 2:**
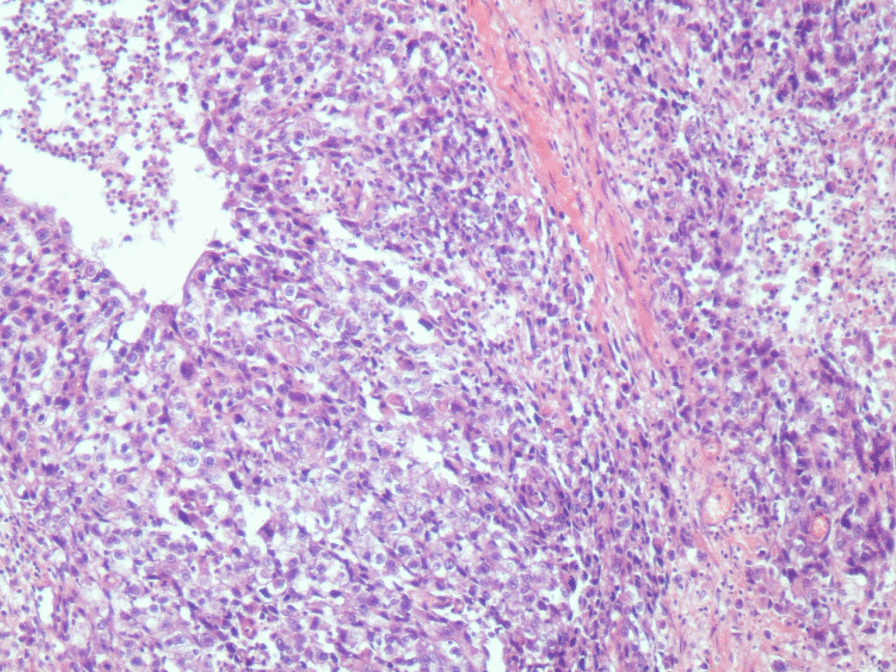
Microscopic examination demonstrating the proliferation of large atypical cells with abundant granular eosinophilic cytoplasm, large atypical nuclei with condensed chromatin and prominent nucleoli (Hematoxylin and Eosin H&E: ×100)

**Fig. 3:**
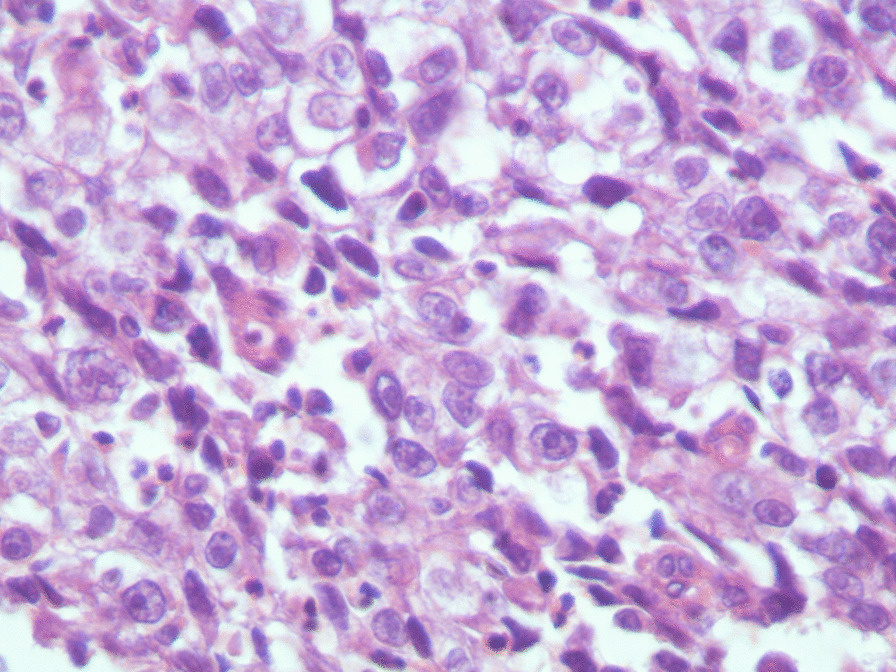
Microscopic examination demonstrating the proliferation of large atypical cells with abundant granular eosinophilic cytoplasm, large atypical nuclei with condensed chromatin and prominent nucleoli (H&E ×400)

**Fig. 4:**
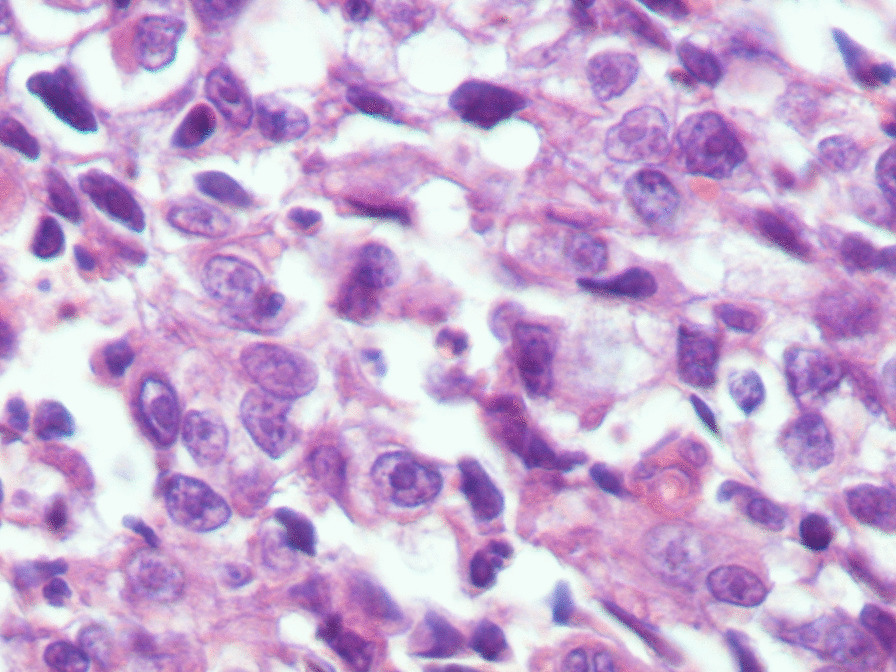
Microscopic examination demonstrating the proliferation of large atypical cells with abundant granular eosinophilic cytoplasm, large atypical nuclei with condensed chromatin and prominent nucleoli (H&E ×600)

**Fig. 5:**
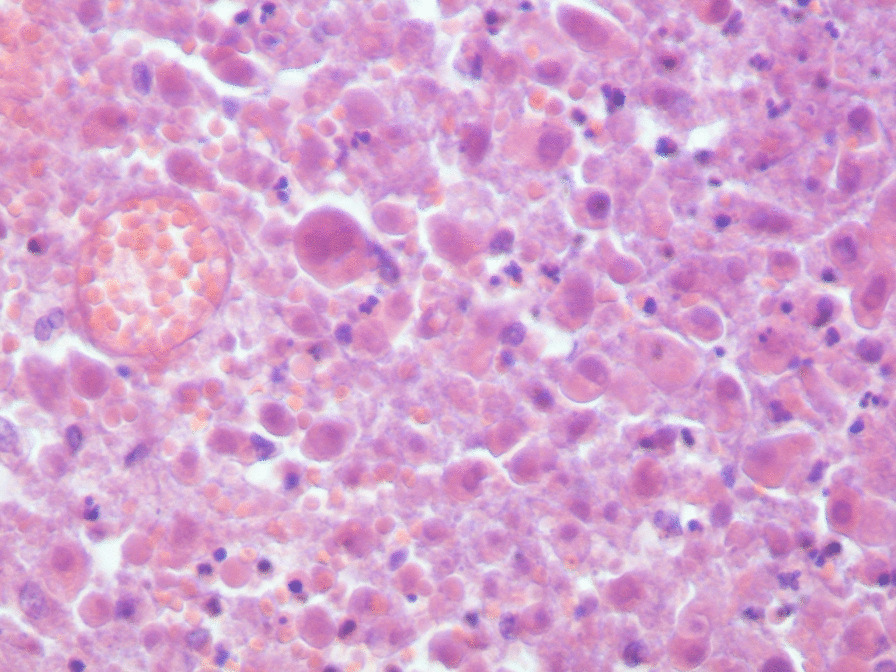
Microscopic examination demonstrating the proliferation of large atypical cells with foci of necrosis (H&E: foci of necrosis)

**Fig. 6:**
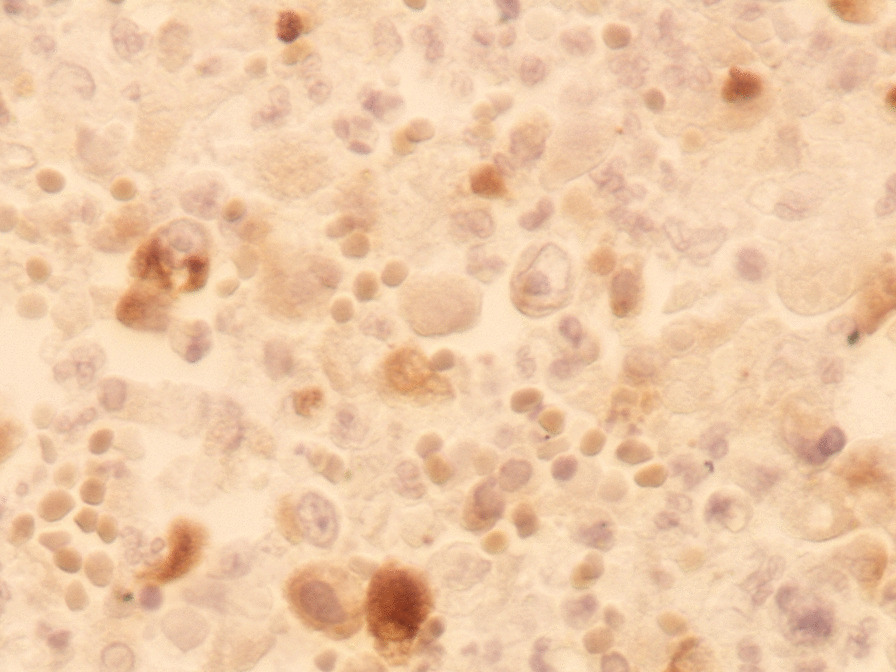
Immunohistochemical examination demonstrating positive expression of androgen receptor AR

**Fig. 7:**
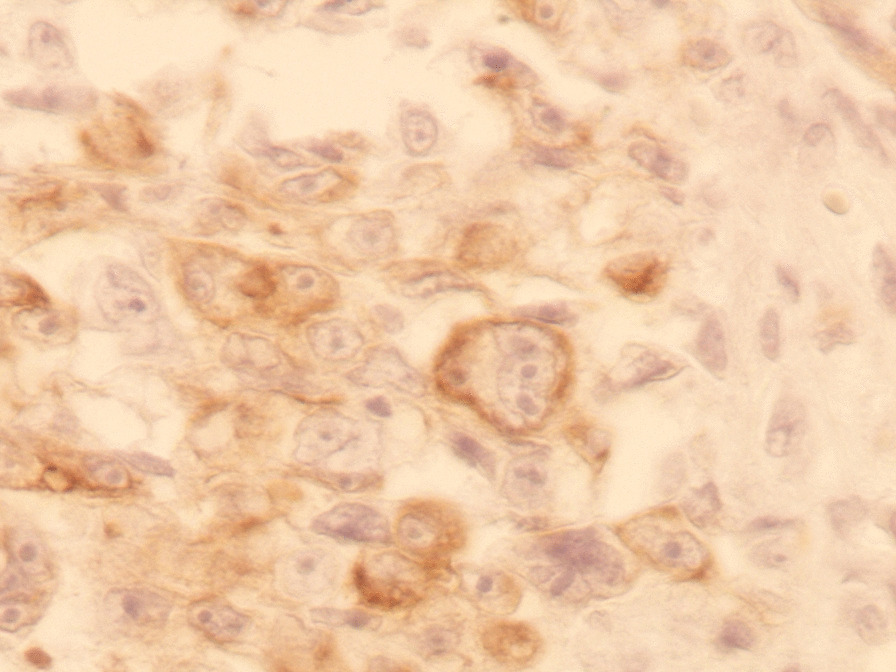
Immunohistochemical examination demonstrating positive expression of GCDFP-15

**Fig. 8:**
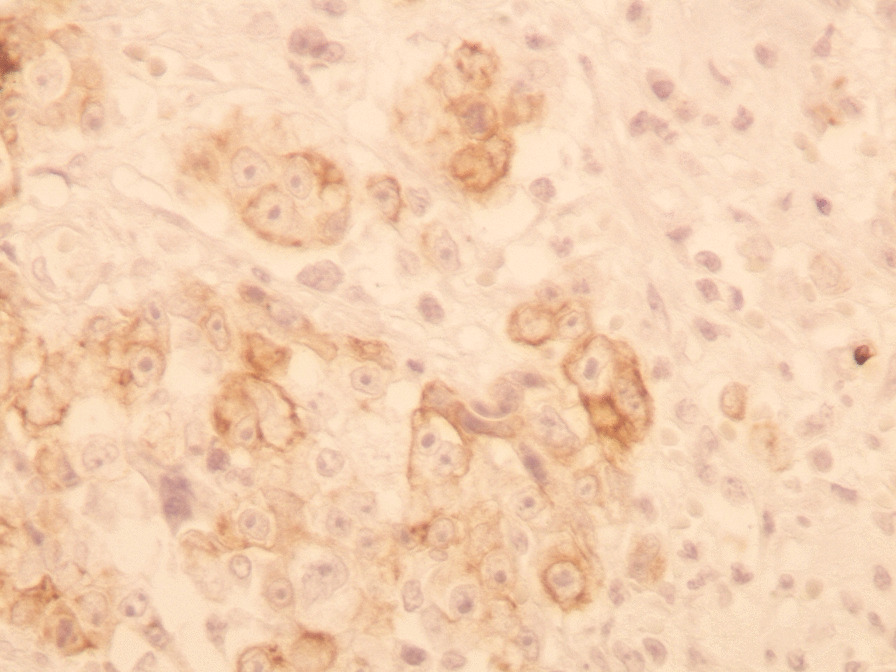
Immunohistochemical examination demonstrating positive expression of cytokeratin CK

**Fig. 9:**
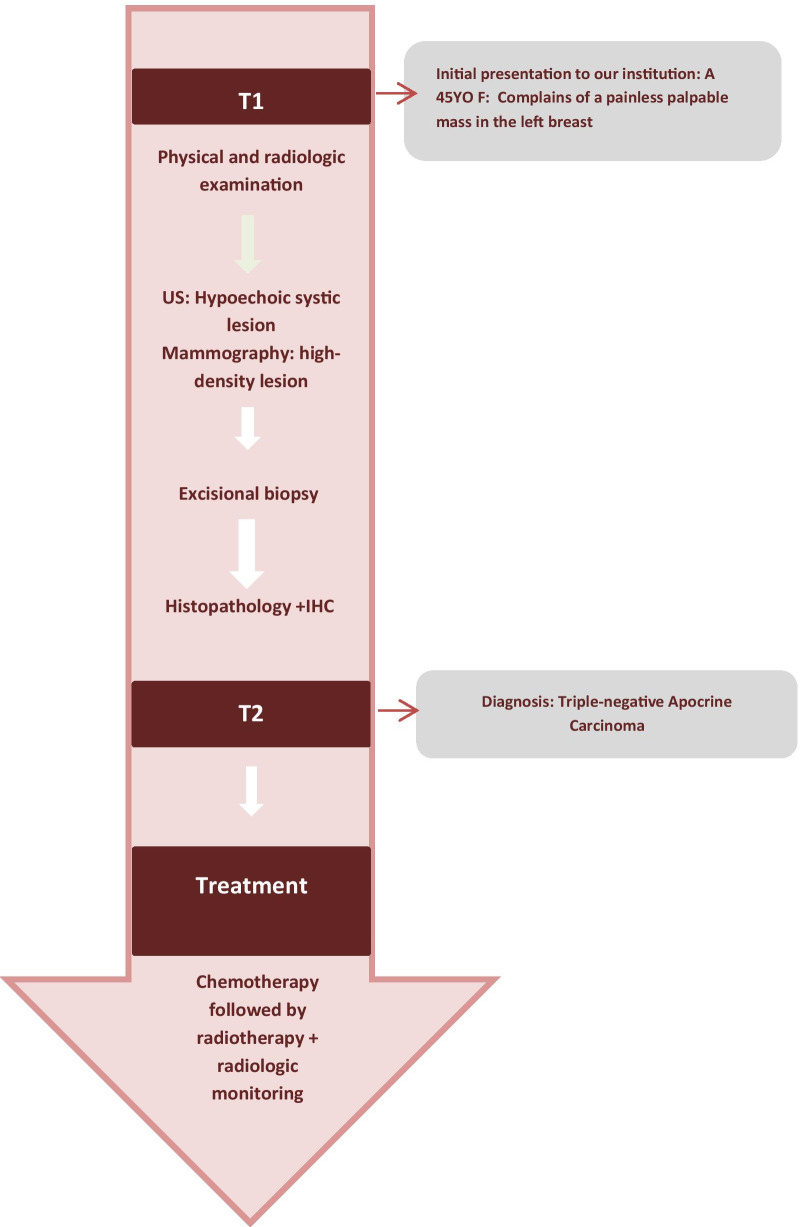
A timeline of the patient’s case

We report a case of a 45-year-old Syrian female who presented to our hospital due to a painless palpable mass in her left breast. The patient is non-smoker and non-alcoholic. Family history was unremarkable as well as medical and surgical history, and the patient mentioned noticing the mass one month prior to her presentation during self-physical examination, with no other symptoms and no previous radiological examinations. On presentation, physical examination revealed a painless palpable mass in her left breast, with no skin changes, and her laboratory findings were within normal limits. Mammographic scanning demonstrated a well-defined high-density mass measuring approximately 3 cm in diameter. Ultrasonography of the breast revealed a well-defined hypoechoic cyst with heterogeneous components (Fig. 1). Abdominopelvic ultrasonography and chest X-ray scanning revealed no other lesions. Following radiological and clinical examinations, an excisional biopsy was.

performed and revealed an irregular-shaped mass measuring 5 × 4.5 cm including the tumor mass with the surrounding tissue of the breast. Cut section demonstrated an ill-defined white-greyish central nodule measuring 3 cm in diameter with central necrosis. Microscopic examination demonstrated the proliferation of large atypical cells with abundant granular eosinophilic cytoplasm, large atypical nuclei with condensed chromatin, and prominent nucleoli, in addition to foci of necrosis (Figs. 2, 3, 4, 5). Primary differential diagnoses included apocrine carcinoma and oncocytic carcinoma. Immunohistochemical examinations revealed positive expression of androgen receptor (AR), Gross cystic disease fluid protein-15 (GCDFP-15), and cytokeratin (CK) (Figs. 6, 7, 8), whereas estrogen receptor (ER), progesterone receptor (PR), and human epidermal growth factor receptor-2 (HER2) were negative. Thus, we confirmed the diagnosis of a triple-negative apocrine carcinoma. Axillary lymph nodes were normal in appearance in clinical and radiological examinations, and no lymph nodes dissection was performed. Later, treatment decision was to schedule the patient for a chemotherapy regimen consisting of Carboplatin and Paclitaxel. The patient is undergoing chemotherapy. She will also be monitored with ultrasonography at periodic intervals of three months for the first two years. A timeline of the patient's case can be seen in Fig. 9.

## Discussion and conclusions

Although apocrine carcinomas were first described by Krompecher et al. [5], the histological criteria to diagnose apocrine carcinomas wasn’t set until Japaze et al. suggested that apocrine differentiation, characterized by the proliferation of large sharply-defined cells with abundant eosinophilic granular cytoplasm, large vesicular nuclei with N/C ratio of 1:2 or more, and conspicuous nucleoli, must be detected in more than 75% of neoplastic cells [6]. Later, Vranic et al. proposed that in terms to diagnose apocrine carcinomas, the aforementioned features must be detected in more than 90% of neoplastic cells with a distinct steroid hormonal profile defined by the negative expression of ER and PR and positive expression of AR as in our case [2]. These strict criteria explain the rarity and difficulty in diagnosing pure apocrine carcinomas which constitute less than 4% of breast cancers, compared to apocrine differentiation which is present in approximately 30% of breast benign and malignant lesions [1].

Apocrine carcinoma is similar in clinical presentation to invasive ductal carcinoma. They both have a predominance in women older than 40 years old as in our case, and presentation varies vary from asymptomatic to the presence of a palpable mass [1, 7]. Our patient presented with a painless palpable mass in her left breast with no skin changes.

Apocrine carcinomas demonstrated disparities in radiological features. Onoue et al. reported two hypoechoic cysts on sonography with papillary projections [8], whereas Gokalp et al. described two invasive apocrine carcinomas presenting as a solid lesion and a complex cyst, respectively [9]. In a study by Seo et, five ACs presented as non-circumscribed irregular-shaped heterogeneous solid masses on sonography [10]. In our case, US scanning revealed a well-defined hypoechoic cyst with heterogeneous components. On mammography, most apocrine carcinoma cases present with microcalcifications. However, Kim et al. reported a case of an AC presenting as an oval nodule without microcalcifications [11].

The main differential diagnoses include oncocytic carcinoma and invasive ductal carcinoma. Invasive ductal carcinoma is similar in architectural growth pattern to apocrine carcinomas. However, the cytological features of apocrine cells are crucial to confirm the diagnosis. Apocrine cells are classified into type A cells which are characterized by abundant granular eosinophilic cytoplasm, and type B cells which have abundant foamy cytoplasm with intracytoplasmic lipids. In our case, most of the cells were of type A. Differentiating apocrine carcinomas from oncocytic carcinomas is more challenging as they both have large strictly defined cells with abundant eosinophilic granular cytoplasm. Therefore, immunohistochemistry is essential. Oncocytic carcinomas could be excluded in our case through the negativity of ER and PR with the positivity of AR and GCDFP-15 [1, 12].

Gross cystic disease fluid protein-15 (GCDFP-15) and Androgen Receptor (AR) are known as THE hallmarks of apocrine differentiation despite their presence in other breast carcinomas. Furthermore, the negative expression of ER, PR, BCL2, and GATA3 also define apocrine differentiation [1, 13]. In our case, we managed to confirm the diagnosis based on the positive expression of AR, GCDFP-15, and CK, with negative expression of ER, PR, and HER2.

HER2/new overexpression was detected in 54% of apocrine carcinoma cases in a study by Vranic et al. [2], whereas HER2-negative apocrine carcinoma is defined as a triple-negative apocrine carcinoma TNAC, which is a rare distinct subtype of triple-negative breast carcinomas TNBCs, characterized by negative expression of ER, PR, and HER2 with positive expression of AR [1, 2]. In a large population-based study by Liao et al. on 19,900 cases of TNBC, apocrine carcinomas were diagnosed in 199 cases (1%) [3]. This highlights an additional peculiar point in our rare case of a triple-negative apocrine carcinoma, alongside being considered –to our knowledge- the first case report from Syria.

Most HER2/neu negative apocrine carcinomas or triple-negative apocrine carcinomas demonstrated EGFR overexpression and vice versa. Also, Vranic et al. detected polysomy of chromosome 7 in several cases of pure apocrine carcinomas [2]. In a study by Farmer et al., activation of AR pathway was detected in apocrine carcinomas [14]. Also, two studies by Naderi et al. demonstrated crosstalk between AR with extracellular signal-regulated kinase pathway ERK, and GCDFP-15 was actively regulated by the aforementioned pathway [15, 16].

Furthermore, in a large genomic sequencing study, Sun et al. demonstrated that PIK3CA was the most predominant mutated gene in triple-negative apocrine carcinomas, with p.H1047R representing the most recurrent mutation. Therefore, PIK3CA inhibitors might represent promising treatments for PIK3CA mutated TNACs. PTEN gene was also highly mutated in the aforementioned study, whereas TP53 mutations were less frequently detected in TNAC cases, in contrast to non-apocrine triple-negative breast cancers [17]. In our case, molecular and genetic studies were not available due to economic restrictions in our country, which increased the challenges in the diagnosis. However, with detailed clinical, histological, and immunohistochemical correlations, we were able to successfully diagnose and manage the patient.

In a large cohort study by Arciero et al., triple-negative apocrine carcinomas TNACs had a significantly better overall survival compared to triple-negative invasive ductal carcinomas TNBCs. Also, their study demonstrated that most TNAC cases were associated with older age, lower T stage, and tumor grade [4]. Similar results were demonstrated by Wu et al. in a large SEER-based study, and in a small limited case–control study by Meattini et al. Studies referred this difference to the overexpression of AR and GCDFP-15 which might be associated with a decreased tumor proliferation and subsequently, a better prognosis [18, 19].

Furthermore, AR expression in apocrine carcinomas could represent a potential target for treating TNACs, although studies regarding the efficacy of anti-androgens are still limited [20]. A study by Gucalp et al. on bicalutamide in patients with triple-negative AR-positive breast cancer reported a 19% clinical improvement on phase 2 clinical trial [21]. Other promising approaches include targeting phosphatidylinositol 3‐kinase oncogene (PIK3CA) as well as inhibiting cyclin-dependent kinases CDK4/6 which are increased in apocrine carcinomas [22, 23]. In a study by Lia et al., TNBCs with residual disease had a better overall survival upon adjustment of conventional chemotherapy, whereas neoadjuvant chemotherapy improved overall survival in TNBC patients with complete pathological response [24]. Therefore, the correct treatment decision is still controversial, and further studies are recommended to assess the correct treatment options. In our case, surgical excision had a crucial role in establishing the correct diagnosis in addition to its importance in the treatment of a non-metastatic apocrine carcinoma. Also, adjuvant chemotherapy of paclitaxel and carboplatin were performed as a first-line method in the treatment of our case of a triple-negative apocrine carcinoma.

In conclusion, although triple-negative apocrine carcinomas are extremely rare neoplasms, they must be considered in the differential diagnosis of breast lesions, and diagnosis must be based on strict criteria through assessing morphological features of apocrine differentiation as well as immunohistochemical examinations. In our manuscript, we aimed to present the first case report of a Syrian female who was diagnosed with this rare malignancy, aiming to highlight the importance of detailed clinical, histological and immunohistochemical correlations despite all circumstances in order to assess the appropriate management.

## Data Availability

Data and material are available on reasonable request from the guarantor and mentor of this study Prof. Alshehabi.

## Abbreviations

AC: Apocrine Carcinoma
TNBC: Triple-Negative Breast Cancer
TNAC: Triple-Negative Apocrine Carcinoma
US: Ultrasonography
CT: Computed Tomography
ER: Estrogen Receptor
PR: Progesterone Receptor
AR: Androgen Receptor
GCDF-15: Gross Cystic Disease Fluid Protein-15
HER2: Human Epidermal Growth Factor Receptor-2
CK: Cytokeratin
H&E: Hematoxylin and Eosin
